# Genetic Determinants of Crop Timing and Quality Traits in Two Interspecific *Petunia* Recombinant Inbred Line Populations

**DOI:** 10.1038/s41598-017-03528-9

**Published:** 2017-06-09

**Authors:** Yufang Guo, Wei-Kuang Lin, QiuXia Chen, Veronica A. Vallejo, Ryan M. Warner

**Affiliations:** Department of Horticulture, Michigan State University, East Lansing, MI 48824 USA

## Abstract

The rate at which plants develop new nodes (development rate) is a major determinant of crop production time, yet the genetic control of this process, including genetic interactions with crop quality parameters, is poorly understood. We employed a modified genotyping-by-sequencing approach and generated genetic linkage maps with 6,291 and 3,297 single nucleotide polymorphisms (SNPs) for the interspecific *Petunia* recombinant inbred line (RIL) population - *P*. *axillaris* × *P*. *exserta* (AE) and *P. integrifolia* × *P. axillaris* (IA), respectively. Comparative mapping between the populations revealed perfect collinearity of marker order but different recombination frequency at the corresponding linkage groups (LGs). Quantitative trait loci (QTL) mapping conducted for development traits and other important quality traits indicated QTL clustered on chromosome 1, 2, 4 and 6 for the AE population and chromosome 1, 2, 5 and 6 for the IA population. Additionally, 209 differentially expressed unique transcripts were identified in shoot apex tissue between fast- and slow-developing RILs, 13 of which mapped to within 1 cM of a development rate QTL. These results will facilitate the identification of novel genes controlling crop timing and quality traits in *Petunia* and highlight the power of using multiple interspecific populations to elucidate genetic determinants of natural variation.

## Introduction

The rate of vegetative node formation (development rate; the inverse of plastochron^1, 2^) strongly influences the timing of flowering for plants grown under inductive conditions, such as the appropriate photoperiod, as flowering time is a function of how many nodes form before the transition to reproductive development occurs, and the rate at which those nodes form^3^. Petunia (*Petunia hybrida*) is an important annual bedding plant typically produced in greenhouses during cold periods of the year in northern latitudes of North America and Europe. Thus, energy inputs for greenhouse heating represent a major cost of production. We previously determined that some accessions of the *P. hybrida* progenitor species, *P. axillaris* (PA) and *P. integrifolia* (PI)^4^, exhibited faster development rates than a broad panel of commercial germplasm, particularly at relatively cool temperatures (14–17 °C), while the wild relative *P. exserta* exhibited development rates equal or inferior to the commercial cultivars across a range of temperatures (14–23 °C^5^). In a *P. integrifolia* × *P. axillaris* F_2_ population, we identified QTL for development rate on chromosomes 1, 2 and 5, collectively explaining 34% of the variation^2^. However, low marker density limits the utility of these QTL for identifying candidate genes controlling development rate. We recently generated reference transcriptomes for *P. axillaris*, *P. exserta* (PE) and *P. integrifolia* comprised of shoot apex, whole seedling, mixed floral development stages, trichome and callus tissue libraries^6^. These transcriptomes, combined with the recently published *P. axillaris* and *P. inflata* genome sequences^7^ vastly improve the sequence resources available to facilitate gene discovery in *Petunia*.

The genetic determinants of vegetative development rate are complex and only partially understood, but appear to involve hormonal regulation and miRNA-regulated pathways. Overexpression of cytokinin oxidase in tobacco resulted in a decrease in cytokinin concentration and a decrease in development rate^8^. Similarly, the arabidopsis *slomo* mutant exhibits reduced free auxin levels and a decreased development rate^9^. Expression of the rice genes *PLASTOCHRON1* and *PLASTOCHRON2*, encoding a cytochrome P450 and a MEI2-like RNA binding protein, respectively^10, 11^, is positively regulated by gibberellin^12^. Mutations at either of these loci result in an increased development rate. The arabidopsis *SQUAMOSA PROMOTER BINDING PROTEIN-LIKE* (SPL) transcription factors *SPL9* and *SPL15* act redundantly to increase plastochron (i.e. decrease development rate), while overexpression of miR156, which reduces expression of 10 *SPL* genes, including *SPL9* and *SPL15* ^13^, decreases plastochron length^1^. Silencing of two clade-VI *SPL* petunia paralogs, *PhSQUAMOSA BINDING PROTEIN1* (*PhSBP1*) and *PhSBP2*, had opposing effects on development rate. Silencing *PhSBP1* decreased development rate while silencing *PhSBP2* increased development rate, possibly through upregulation of *PhCNR*, a petunia homolog of the tomato *SPL* gene *LeSPL-COLORLESS NON-RIPENING* ^14^. Mutations in *SERRATE*, an arabidopsis zinc finger protein involved in miRNA biogenesis and primary miRNA processing^15–17^ result in reduced development rate. These results indicate that the control of development rate is complex, and much remains to be elucidated.

Understanding the genetic determinants of development rate could facilitate breeding of cultivars with increased development rate, particularly at lower temperatures, which could reduce energy inputs and costs of production. However, if development rate is to be manipulated to facilitate reductions in crop production time, it is critical that crop quality not be compromised in the faster-developing genotypes. It is therefore important to understand potential genetic interactions between development rate and crop quality traits such as branching and flowering. Herein, we describe the development, characterization and utilization of two interspecific F_7_ *Petunia* recombinant inbred line (RIL) populations, *P. integrifolia* × *P. axillaris* (the “IA” population), and *P. axillaris* × *P. exserta* (the “AE” population), for quantitative trait locus (QTL) mapping of development rate and other crop timing and quality traits. Multiple QTL for development rate were identified in each population, and most were population-specific. Additionally, we identified differentially expressed genes in shoot apex tissue between fast- and slow-developing IA RILs. These results will facilitate gene discovery to further elucidate genetic control of development rate and other crop timing and quality traits in petunia.

## Results

### Crop timing and quality traits in two Petunia recombinant inbred line populations

Seven traits for the *P*. *axillaris* × *P*. *exserta* (AE) population and sixteen traits (including the seven measured for AE) for the *P*. *integrifolia* × *P*. *axillaris* (IA) population were measured under three temperatures (14, 17 and 20 °C) (See Materials and Methods for trait descriptions). Transgressive segregation was observed for all traits in both RIL populations (Supplementary Tables S1 and S2), similar to previous reports for interspecific F_2_ populations in petunia. The crop timing related traits development rate (DRate), days to anthesis of the first flower (DTA) and node number below first flower (Nodes) consistently exhibited greater population range for the IA than the AE population. Temperature influenced the mean values of both populations for the crop timing traits DRate and DTA, as well as some quality traits. Broad-sense heritability (H^2^) estimates were lower for all traits than previously reported for the F_2_ generation of these populations^5^. DRate for both populations across the three temperatures was significantly and positively correlated. Similarly, DRate was positively correlated with Nodes, and negatively correlated with DTA in the IA population. For the AE population, this correlation was similarly negative at 14 °C, but was not significant at 17 and 20 °C (Supplementary Tables S3 and S4). This suggests that selecting for a fast development rate could decrease time to flower, particularly at cooler temperatures, even though plants may produce more nodes before flowering.

Overall, 63.8% of trait correlations (across temperatures) were significant for the AE population (Supplementary Table S3). Each trait was significantly correlated with at least 11 (42%) other traits in the AE population, indicating pleiotropy or linkage disequilibrium in the genetic mechanisms of some of the traits in this study^18^. Nodes had the highest incidence of correlation with other traits across temperatures. For the IA population, 43.2% of trait correlations (across temperatures) were significant (Supplementary Table S4). LWid (width of the third leaf below the flowering node), Nodes, and DRate were among the traits with the higher percentage of correlation with other traits.

### Linkage map construction from GBS

#### Constructing SNP-based bin maps for both populations

The tunable genotyping by sequencing (tGBS) generated more than 3 million raw reads for *P*. *axillaris* (PA), 2 million reads for *P*. *exserta* (PE), 1 million reads for *P*. *integrifolia* (PI) and about 216 million and 250 million raw reads for the AE and IA populations respectively. After trimming low quality bases (0.8% to 1.2% from each population). 56.3%–73.5% of the trimmed reads were uniquely mapped to the reference genome of *P*. *axillaris* ^7^ for SNP (Single Nucleotide Polymorphism) detection. After the SNP filtration steps, 6,582 and 5,228 SNPs were identified for the AE and IA populations, respectively. Draft genetic linkage maps for AE had 7 LGs (linkage groups) with 6,291 SNPs (96% of total SNPs) for the AE population and 14 LGs with 3,297 SNPs (63% of the total SNPs) for the IA population.

**Figure 1:**
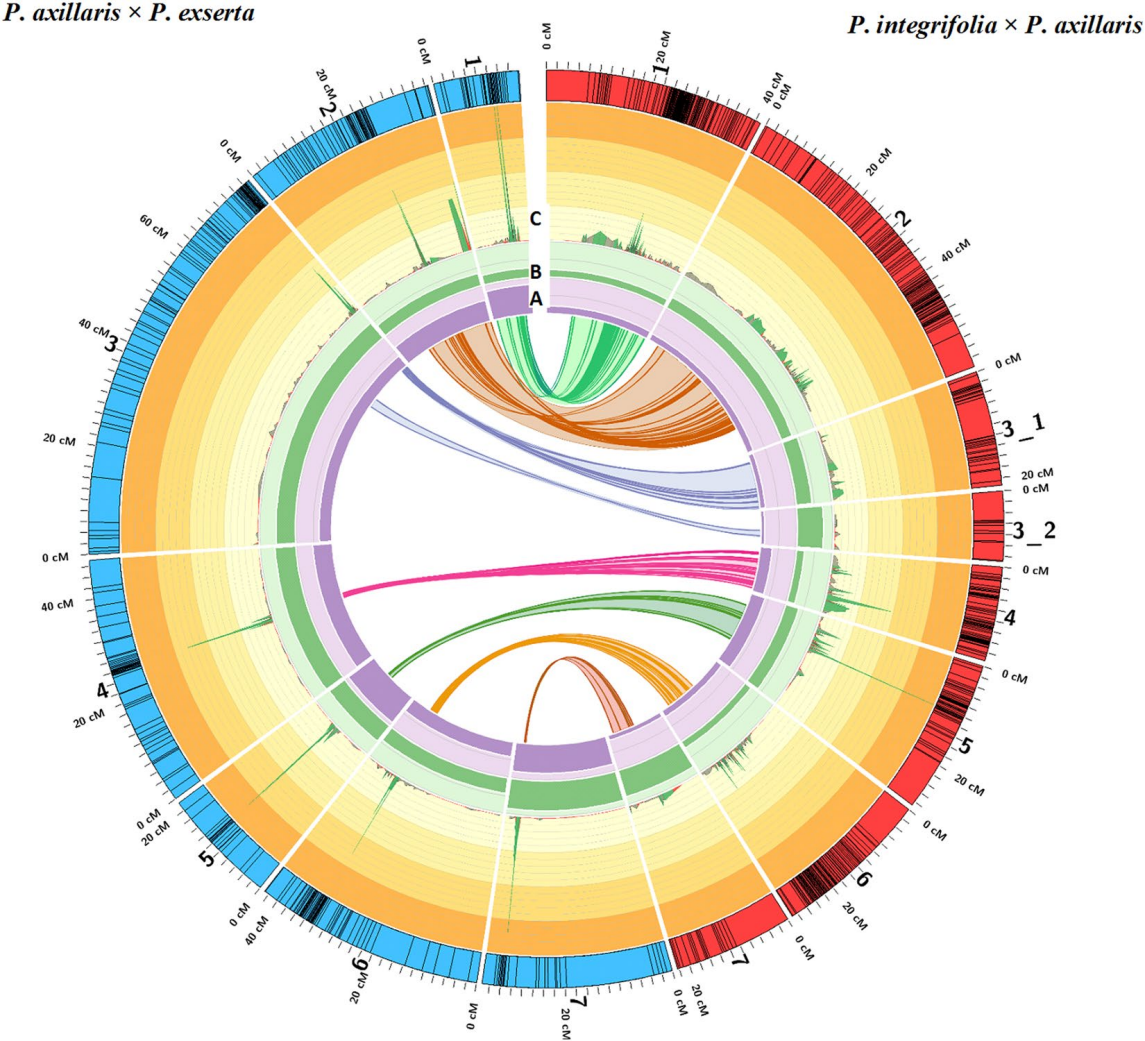
Genetic map of *P*. *integrifolia* × *P*. *axillaris* (IA) and *P*. *axillaris* × *P*. *exserta* (AE) populations, and the synteny between the linkage groups. Rings represent: (**A**) Average number of markers per bin; the darker purple shade indicates range from 0 to 31; (**B**) Average distance (in cM) between bins (or between markers for the IA map); the darker green shade indicates ranges from 0 to 1.6 cM; (**C**) SNP frequency; each different shade of yellow indicates a 1% increase, the minimum percentage shown is 0.03% and the maximum percentage shown is 4%.

SNP binning yielded 330 bins for the AE population and 457 bins for the IA population. In addition, SSR (Simple Sequence Repeat) and CAPS (Cleaved Amplified Polymorphic Sequences) markers were added to the IA population to connect the fragmented linkage groups^6^. The reconstructed AE bin map had seven LGs with a total genetic distance of 277.08 cM (centimorgans). Genetic length ranged from 15.99 to 77.37 cM, and average marker interval ranged from 0.41 to 1.36 cM. The IA map had eight LGs from 12.94 to 59.83 cM, and a total length of 233.01 cM. The average marker interval ranged from 0.31 to 1.24 cM (Table 1 and Fig. 1, Tables S5 and S6).

**Table 1:** Summary of the linkage maps for the *P*. *axillaris* × *P*. *exserta* (AE) RIL population and the *P*. *integrifolia* × *P*. *axillaris* (IA) RIL populations.

AE					IA					
Linkage Group	No. Bins	No. mapped SNPs	Map Length, cM	Average marker density, cM	Linkage Group	No. Bins	No. mapped SNPs	No. other type markers	Map Length, cM	Average marker density, cM
1	40	1032	15.99	0.41	1	125	756	10	41.04	0.31
2	67	1238	36.60	0.55	2	111	566	8	59.83	0.51
3	92	945	77.37	0.85	3_1	32	125	0	21.43	0.69
					3_2	11	27	1	12.94	1.18
4	52	858	47.17	0.92	4	52	519	3	17.58	0.33
5	26	731	21.60	0.86	5	43	529	7	28.78	0.59
6	64	806	42.97	0.68	6	93	503	3	29.13	0.31
7	27	681	35.37	1.36	7	15	55	4	22.28	1.24

The average inter-marker distance on each LG ranged from 0.46 (chromosome 1) to 1.79 cM (chromosome 7) for the AE map and from 0.30 cM (chromosome 1 and chromosome 6) to 1.23 cM (chromosome 7) for the IA map (Table 1 and Fig 1). Therefore, chromosome 1 had higher resolution than chromosome 7 in both populations. The average marker density was 0.49 and 0.76 cM for the IA and AE maps, respectively. The average number of markers per bin ranged from 2.5 (chromosome 3_2) to 12.3 (chromosome 5) for the IA population and from 10.3 (chromosome 3) to 28.1 (chromosome 5) for the AE population (Table 1 and Fig. 1, Tables S5 and S6).

#### Synteny between the IA and AE populations

Extensive synteny was identified between the populations (Fig. 1, Supplementary Table S7). Synteny between LGs could be represented by a total of 260 SNPs at the same physical location in both populations (Supplementary Table S7). Some of the SNPs were located in sub-bins, yet the exact SNP within the sub-bin was unclear, which may cause the synteny between bins and sub-bins to be ambiguous, although the sub-bins tended to co-localize/cluster on the same LGs.

The entire LG for chromosomes 3, 4, and 6 for the IA population corresponded to only a small portion of the same chromosomes for the AE population (Fig. 1). Within the syntenic region, assuming the same physical distance was covered for both maps (based on the corresponding SNPs), the genetic distance was overall larger for the IA than the AE population, indicating the higher recombination frequency in the IA population within this region. However, some AE LG fragments outside the syntenic regions are completely unrepresented in the IA map, leading the IA map to have overall less genomic coverage.

#### Segregation distortion for AE and IA linkage maps

For the IA population, 85.5% of the markers (bins) showed segregation distortion, and 89.1% of the distorted markers skewed towards *P*. *axillaris*. Chromosome 2 and chromosome 3_2 had 42.0% and 58.3% of the markers displaying segregation distortion, while the rest of the chromosomes had 100% segregation distortion. All of the distorted markers skewed towards *P*. *integrifolia* were located on chromosome 2. For the AE population, 51.1% of the bins showed segregation distortion and over 70% of the skewed bins were skewed towards *P*. *exserta*. Both chromosome 2 and 6 had 100% skewed markers, with 98.5% (chromosome 2) and 100% (chromosome 6) skewed towards *P*. *exserta*.

### QTL identification for crop quality traits in petunia

#### Overview of QTL for both RIL populations

**Figure 2:**
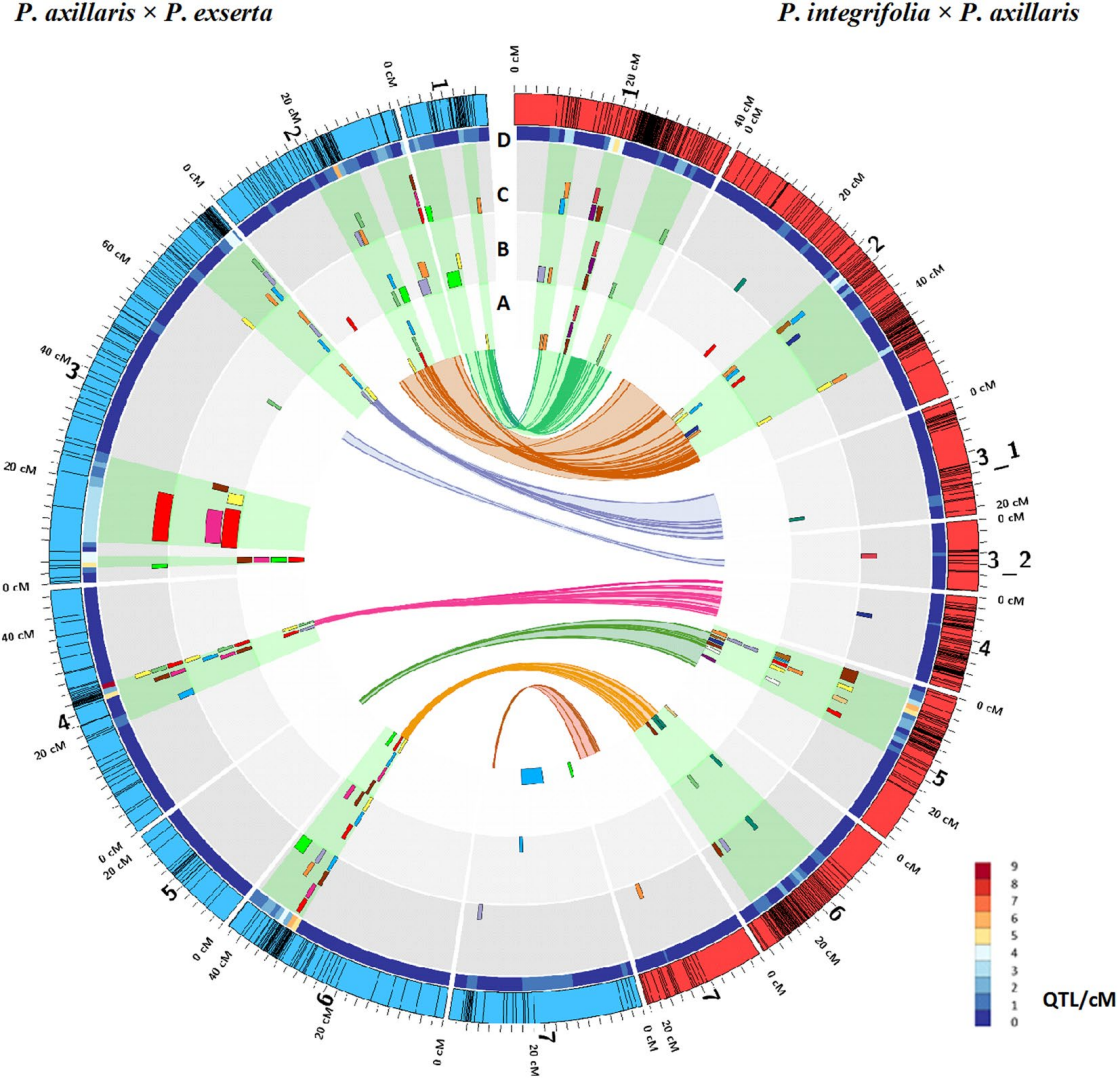
Overall QTL summary for *P*. *integrifolia* × *P*. *axillaris* (IA) and *P*. *axillaris* × *P*. *exserta* (AE) populations. Rings represent QTL detected at 14 °C (**A**), 17 °C (**B**), and 20 °C (**C**). Each color represents a different trait, and areas highlighted in green represent QTL rich regions. (**D**) Heat map showing the overall QTL density across each linkage group.

Seven traits were common between the two populations (Nodes, DTA, FIDiam- diameter of first flower, LLeng - length of the third leaf below the flowering node, LWid, DRate, and Height - height to the first flower node). For the AE population, DRate was calculated at two time points and therefore was divided into three traits (e.g. at 14 °C: DRate14_1, DRate14_2, and DRate14_all). The corresponding DRate for the IA population is DRate_all for the AE population. Overall, 26 to 29 QTL for these traits were detected at each temperature for AE, and 12 to 14 QTL were detected for IA (Fig. 2, Table S8). The number of QTL for each trait ranged from 1 to 5 with a mean of 3.1 for AE and from 1 to 4 with a mean of 2 for IA. For the common traits, utilizing two populations with different backgrounds could provide a better characterization of the nature of quantitative genetic variation. In addition to genetic background, the larger number of QTL per trait for the AE population may result from phenotyping more RILs for the AE than IA population, as larger populations can enable the detection of smaller effects QTLs, while QTL effects are generally overestimated in small populations^19^.

For the crop timing traits DRate, DTA, and Nodes, in the AE population, QTL were detected for all three traits under all temperatures. In the IA population, QTL were detected for DTA and DRate under all three temperatures, and were detected for Nodes at 17 °C and 20 °C (Fig. 2, Table S8). For Nodes in the AE population, chromosomes 4 and 6 had relatively larger effect, stable QTL under all three temperatures, while chromosomes 2 and 3 had QTL with fluctuating locations, which might indicate QTL affected by micro-environment. QTL for DTA were detected consistently across temperature on chromosomes 2 and 4, while chromosome 3 had a smaller effect DTA QTL at 17 °C and 20 °C with shifted locations. The DRate measurements were divided into three time intervals, and QTL were detected for at least one interval at each temperature. Similar to Nodes, stable, larger effect QTL for DRate was consistently identified on chromosome 4 and 6 for most time intervals. Some QTL could only be detected under certain temperatures, suggesting conditional expression of some of the genes controlling development rate. Overall, chromosome 4 and 6 harbored larger effects QTL that were located at a very narrow region for crop timing traits, all alleles that could increase development rate were from *P*. *axillaris* (Fig. 2, Table S8). For the IA population, QTL for DRate were only detected on chromosomes 1, 5, and 6. The alleles that improve DRate were from *P*. *axillaris* on chromosome 1 and 6 and from *P*. *integrifolia* on chromosome 5. The QTL detected on chromosome 1 were located at almost the same location under all three temperatures.

For the same traits, QTL direction from both populations indicated contribution to the traits could come from different species (parental lines). For instance, for the AE population, chromosome 3 had larger effect QTL at approximately the same locations under all three temperatures for the crop quality traits FlDiam, LLeng and LWid. Of all QTL detected for these three traits, the alleles contributing to the increase of these traits were from both parents, but the majority of the alleles under the “stable” QTL were from *P*. *exserta*. Interestingly, the genetic controls for these traits in the IA population seemed to be different from the AE population, as the majority of the QTL detected were located at different chromosome locations than in the AE population. The alleles that contributed to the increase of the traits were from both *P*. *integrifolia* and *P*. *axillaris* and chromosomes 1, 2, and 4 harbored most of the major effect QTL (Fig. 2, Table S8).

In addition, seven traits were only recorded in the IA population ([LNMS - the leaf number on the primary shoot, Branch- the number of lateral branches > 8 cm in length, HMS - the height of the main stem (to the apical meristem) at flowering, HSB - the height from the soil surface to the flowering side branch, FIBudPS - the number of visible flower buds on the main stem (for plants where the first flower opened on the main stem), FIBud - the total number of visible flower buds, and FIBranchNum - the number of lateral shoots with visible flower buds]. At 14 °C, eleven QTL were identified for these traits, while at 17 °C six QTL were identified for five of the traits, and at 20 °C nine QTL were identified for six of the traits (Table S9). Out of the 26 total QTL, 23 were located on chromosome 1, 2, 5 or 6. For 18 out of the 26 QTL, the *P*. *axillaris* allele contributed increase of the trait. A major QTL for Branch was detected on chromosome 1 that explained 20% to 30% of the phenotypic variation. Similarly, a major QTL on chromosome 1 was detected for FIBranchNum under all three temperatures. The QTL location for Branch and FIBranchNum were within 3 cM on chromosome 1. The alleles that contributed to the increase of the traits came from *P*. *axillaris* in both cases (Table S9).

#### QTL clustering on the linkage maps of both populations

The QTL identified in this study were concentrated in specific regions on some chromosomes. For the AE population, most QTL were distributed across chromosomes 1, 2, 3, 4, 6 and 7. Almost all QTL detected on these chromosomes were clustered into distinct regions within less than 5 cM, except QTL on chromosomes 3 and 7, where the QTL were located on regions with lower marker density. For the IA population, QTL were identified mainly on chromosomes 1, 2, 5 and 6. Similarly, QTL were located in narrow regions along each chromosome (Fig. 2, green highlighted part). For the AE population, for instance, the region from 25 to 28 cM on chromosome 4 had 15 QTL, with the highest QTL density of 10 QTL/cM. For the IA population, a region on chromosome 5 had 18 QTL, with the greatest QTL density of 6 QTL/cM (Fig. 2, heat map part).

In the AE population, for the QTL rich regions ( ≥ 5 QTL/cM) on chromosomes 2, 3, 4, and 6, the SNPs within these regions comprised 45.0%, 0.003%, 34.5% and 74.7% of the total SNPs on that linkage group (Table S10). The regions with higher QTL density largely overlapped with the high SNP frequency regions, which could represent peri-centromeric regions for chromosome 2, 4 and 6. Conversely, the region with the highest QTL density on chromosome 3 only had three SNPs. However, the region with the second highest QTL density on chromosome 3 overlapped with a putative sub-telomeric region, which contained 70.4% of the total SNPs in the linkage group. These regions of suppressed recombination likely represent large physical distances, which could complicate the identification of candidate genes under the QTL.

We further compared the QTL from both populations that are located on corresponding chromosomes for the common traits (Table S11). For some QTL, the alleles that contributed to the increase of the trait came from different parents in each population, so these two are potentially not the same QTL. Only QTL with the allele contribution from the same direction were further investigated for overlap by identifying the corresponding SNP locations (Table S11). In general, no strong overlap was detected for most of the QTL identified by multiple QTL mapping model (MQM) mapping with 1-LOD supporting intervals with corresponding SNPs at the same location, but more overlapping positions may be identified by increasing the LOD interval or through alternative mapping methods (such as interval mapping).

### Identification and mapping of differentially expressed genes for development rate in the *P*. *integrifolia* × *P*. *axillaris* population

Around 83% to 89% of the trimmed Illumina reads were mapped to the reference transcriptome of *P*. *axillaris* ^6^. A total of 209 unique transcripts were differentially expressed between fast- and slow-developing individuals in the IA population (Fig. S1, Table S11). These differentially expressed genes (DEGs) were further placed onto the IA linkage map if they were within 1,000,000 bp of the SNPs in the IA map. Overall, 135 DEGs could be placed to IA map under this criterion. No overlaps were found between the current DEGs and the petunia homologous transcripts of plastochron-related genes previously identified^6^.

**Figure 3:**
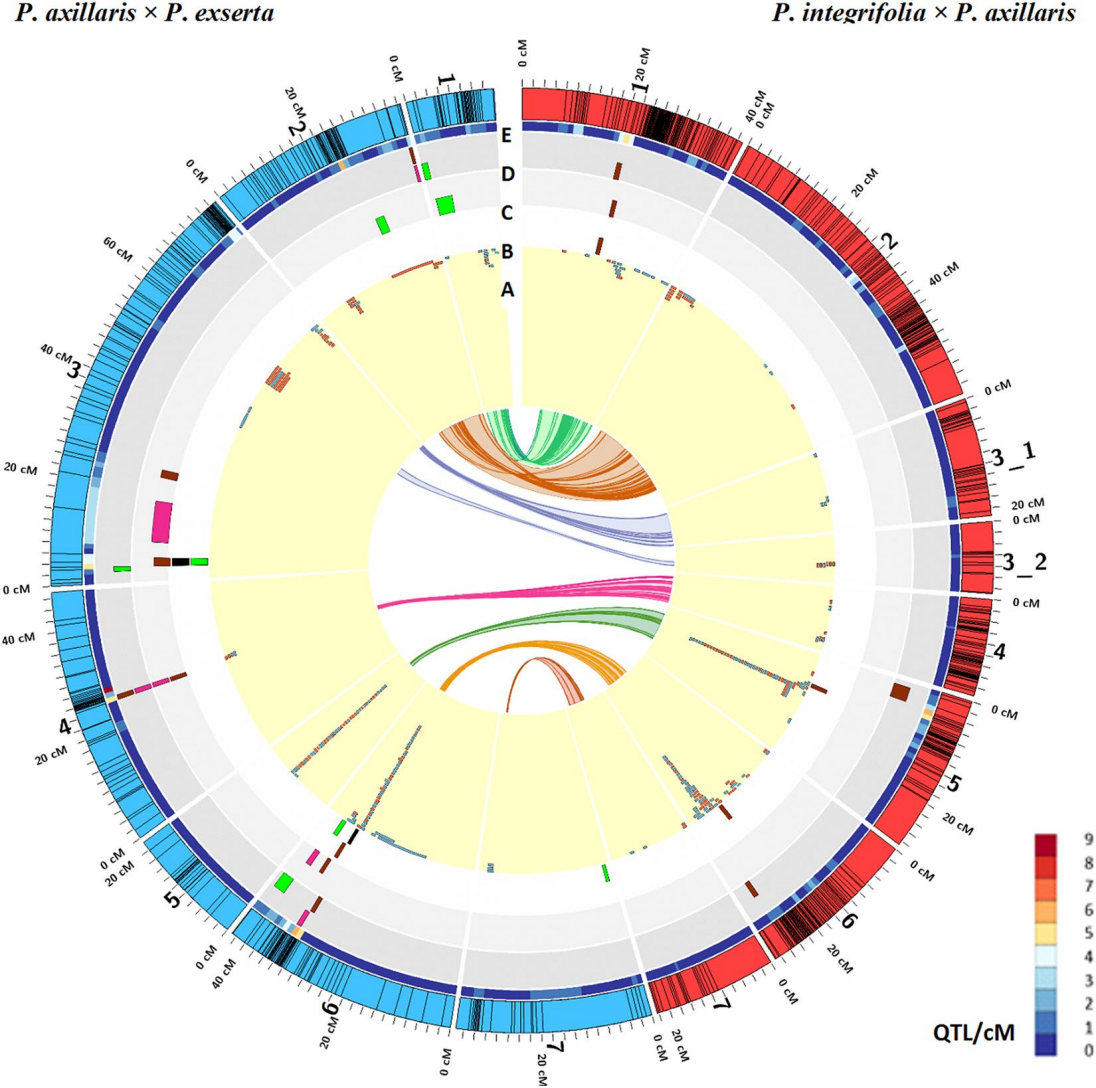
QTL detected for development rate and the differentially expressed genes between “fast” and “slow”-developing RILS on the linkage groups of *P*. *integrifolia* × *P*. *axillaris* (IA) and *P*. *axillaris* × *P*. *exserta* (AE) populations. Rings represent: (**A**) Differentially expressed IA genes mapped to each population; the genes highlighted in red and blue are up-regulated and down regulated genes, respectively. QTL for development rate identified at 14 °C (**B**), at 17 °C (**C**) and 20 °C (**D**). (**E**) Heat map showing the overall QTL density along each linkage group.

Several unique features were detected regarding the DEG locations. First, these genes tended to “stack up” at very narrow genetic regions mainly on chromosomes 5 and 6 for both populations, where QTL for DRate were detected. Some of these had higher expression in *P*. *axillaris* and some had higher expression in *P*. *integrifolia*. Second, the DEGs were not always located in the QTL-rich regions. For the IA population, DEGs tended to be within regions with high SNP frequency and, therefore, suppressed recombination (Figs 1 and 3). But the QTL rich regions did not overlap the suppressed recombination regions in the IA population. For the AE population, the DEGs on chromosome 5 did not correspond to any QTL, but were located at the putative centromeric region with suppressed recombination, while the DEGs on chromosome 4 and 6 were located at QTL rich regions with suppressed recombination. The majority of the DEGs did not map exactly to the QTL location, but were close to these regions. Out of the 209 unique transcripts, 13 were located within 1 cM of the QTL regions on chromosomes 5 and 6 for the IA population (Table S12). Some of the DEGs had functions involved in vegetative development. For example, one transcript (Locus_29838_Transcript_1/1_Confidence_1.000_Length_1480) has a significant similarity with amide-linked conjugates of indole-3-acetic acid (IAA) in Arabidopsis, which is a putative storage or inactivation form of the growth hormone auxin^20^. Another transcript (Locus_12715_Transcript_1/6_Confidence_0.538_Length_1819) has significant similarity with BRIZ2, which has been reported as required for seed germination and post-germination growth in *Arabidopsis thaliana* ^21^. Further research is needed to elucidate the genes underlying the QTL responsible for DRate.

## Discussion

Our study used tGBS and generated high density linkage maps from two petunia interspecific populations. The total map lengths in this study were shorter than previously reported *Petunia* F_2_ or BC_1_ genetic linkage maps^6, 22–24^. The map resolution was greatly improved compared to previous reports. Synteny blocks with the IA F_2_ showed most of the F_2_ LGs only comprised a portion of the corresponding RIL linkage groups^2^, indicating the latter had higher genome coverage. However, this may be because of insufficient marker coverage on the previous maps partially due to marker availability. In general, F_2_ and BC_1_ populations have only one cycle of meiosis, while RILs have undergone several cycles of meiosis, which enables more frequent recombination. Therefore, greater genetic mapping resolution will be achieved in using RIL populations. The length between the two RIL maps in this study may come from the difference of recombination frequency at specific chromosomal regions influenced by the genetic background of different species^23^. For some plant species, the recombination frequency tends to be overestimated partially due to excess heterozygosity in RIL populations, which can cause the genetic map expansion^25^. The SNP filtering and the representative genotyping from the SNP-bin in this study seemed to compensate some of the map expansion by eliminating some heterozygous genotype/individuals. In the long run, using a more robust statistical model for genetic linkage mapping could retain more genetic information in the map and/or may enable a more accurate genetic map estimation. The exact order of the SNPs within the bins and sub-bins could be further refined by increasing the genetic resolution or with the further improvements of the current petunia genome sequence. In this study, SNP frequencies were calculated as the percentage of total mapped SNPs within each bin. A high SNP frequency indicates a large number of SNPs co-segregating within a short genetic distance, although recombination between bins in these regions was also higher. Further comparison of the genetic map with a physical map is needed to transform the genetic recombination rates into physical distances. Considering the number of co-segregating SNPs, these regions may represent suppressed recombination compared to other regions on the linkage groups, and could represent pericentromeric or peritelomeric regions.

Similar to previous study in petunia using interspecific or intraspecific mapping populations^22, 23, 26, 27^, in this study, perfect collinearity of marker order was retained, but different recombination frequency was found between the two interspecific maps. There are several possible reasons to explain the difference in recombination frequency and map coverage between the two maps. First, fewer SNPs were used for the IA map, which could indicate that more SNPs/RILs with a higher percentage of heterozygosity were excluded due to a higher percentage of heterozygosity in the IA population. It might be worth further investigation to monitor the change in residual heterozygosity during the progression of generations by mapping the SNPs outside the current IA map in the F_2_ and early generation RILs. Second, the earlier divergence between *P*. *axillaris* and *P*. *integrifolia* could cause some genetic incompatibility between the species. A previous survey for SNPs from transcriptome assemblies of these three species identified that the number of SNPs between *P*. *axillaris* and *P*. *integrifolia* was 2.85 times of those between *P*. *axillaris* and *P*. *exserta* ^6^. Even though, it remains unclear why the reduced IA LG coverage was found only on certain chromosomes. In petunia, significant differences in genetic recombination frequency were previously observed in the interspecific crosses *P*. *exserta* × *P*. *parodii* and *P*. *axillaris* × *P*. *inflata* ^22^ and in intraspecific crosses among *P*. *hybrida* accessions^27^. In other crops such as maize, significant variation of recombination rate in intraspecific crosses was observed at the whole genome, chromosome, and intra-chromosome level^28^. The difference in recombination rate might have several reasons from previous reports: genome structural variation between species, such as heterochromatic variation that can be seen at karyotype level^29^; sequence level differences, such as repetitive element content, presence-absence or copy number variants, which might contribute to the degree of homology between paired chromosomes^30^; and the presence of a genetic modulating system “Rm” as a gene or a gene complex affecting the recombination rate as a whole^31^. Similarly, we also noticed that the order of the shared markers was mostly the same between the two populations^22^, indicating no chromosome rearrangements between the species.

High frequency of segregation distortion has also been observed for other inter- and intraspecific crosses in *Petunia* ^22, 30^. For example, the skewed “transmission ratio distortion” toward *P*. *integrifolia* on chromosome 2 was found in a (*P*. *axillaris axillaris* N × *P*. *integrifolia inflata* S6) × *P*. *integrifolia inflata* S6 backcross population^24^. A previous report in tomato also found different distortion patterns and directions for two interspecific populations, indicating species-specific locus interactions^22^. Similarly, we found that the segregation direction and the distorted marker location were different in the two populations, which may also suggest species-specific interactions. Currently, there are several mechanisms that can explain segregation distortion, including gametic selection and zygotic selection^32, 33^. Our previous report of low fertility between *P*. *integrifolia* and *P*. *axillaris*, and low self-fertility of the F_1_ hybrids might be an indication of gametic selection and zygotic selection resulting from the greater genetic distance and self-incompatibility^5^. Self-incompatibility has been reported to cause segregation distortion in other Solanaceae crops, such as tomato and potato^34^. In our study, *P*. *integrifolia* was reported as self-incompatible^35^, which is thought to be controlled by S-RNase and pollen-S at S-locus, located at chromosome regions suppressed for recombination. Although there are both self-compatible and self-incompatible accessions of *P*. *axillaris*, this study used a self-compatible accession^36^. In the IA map, we suspect the S-locus could be located chromosome 2, where the majority of the alleles came from *P*. *integrifolia*, or on chromosome 3, where the suppression of recombination inhibited the linkage between the two separate linkage groups. However, the S-locus was not located on genomic scaffolds that harbor the SNPs comprising the IA linkage map^7^. Previous research with computer simulations indicated that distorted markers will not have a great effect on the position and effect estimation of QTL (quantitative trait loci)^37^. The distortion can even sometimes benefit the detection of linked QTL, because of the higher genetic variants from the distorted markers^38^. For large-size mapping populations, their effects can be ignored^37^.

In this study, we detected the progress of segregation distortion during the population development. A previous study with F_2_ population from *P. integrifolia* × *P. axillaris* reported segregation distortion detected along the entire linkage group for chromosomes 4, 6, and 7, all skewed toward *P*. *axillaris* alleles. Here, the RIL population with the same parental lines had increased segregation distortion with more markers/larger genetic regions skewed towards *P*. *axillaris* during the inbreeding process. In other crops such as tomato and rice, increasing segregation distortion with the progression of selfing has also been reportede^39, 40^. Overall, the progress of segregation distortion represents the cumulative of selection pressure from both genetic and environmental factors and could be related to artificial sampling/unintentional selection and natural selection of many generations during the process of population development^41^. The chromosomal regions over representing one parental species may be associated with a selective advantage during the population development.

The broad sense heritability estimation here was overall lower than previous reports for F_2_ populations^2, 5^. In general, the power to detect small-effect QTL was low for traits with lower heritability, but the major QTL should still be detected. The lower H^2^ could mean that, in the RIL populations, the residual variance was high. The linkage mapping indicated segregation distortion on both populations, which might be from self –incompatibility or high genetic distance between the parents. Therefore, even after several generations of selfing, the self-incompatible nature will still leave a higher percentage of residual heterozygosity, which can increase residual variance, However, compared with previous studies, this study is of broader scope by detecting QTL under a range of temperatures, which facilitates identification of common QTL under sub-optimal temperature settings and/or small effect environment-specific QTL.

QTL for Drate has been previously mapped with the *P*. *integrifolia* × *P*. *axillaris* F_2_ population, and three QTL were detected on chromosomes 1, 2 and 5, respectively^2^. This study also identified QTL for Drate on chromosome 1 and 5, indicating that the QTL on these chromosomes might be major QTL not affected largely by the H^2^ from population type (F_2_ vs. RIL) and size change. We found QTL for Nodes were only detected on chromosomes 2 and 5, and alleles from *P*. *integrifolia* contributed to an increase in Nodes on chromosome 5. QTL for DTA were detected on chromosomes 1 and 6, and all alleles that increased DTA were from *P*. *integrifolia* (Table S8, Fig. 2). The population-specific QTL likely represent species-specific alleles. Both populations share a common parent, if for the same traits the QTL contributed by the common parent were only detected in one population, it was likely due to the segregation of genetic variation with the other parents. Similarly, QTL mapping for trichome density from four RIL populations of *Arabidopsis thaliana* identified “lineage specific alleles” for population-specific QTL^42^. Therefore, the above results underscores that using multiple populations can improve understanding of the genetic architecture of natural variation.

We found that the QTLs detected tend to be clustered in both populations. One possible reason for the high QTL density is that the QTL detected for some traits (Height, DTA, and Nodes for AE population) were located at the same or similar locations across temperatures. The co-localization of QTL for different traits may suggest pleotropic regions/genes. Hypothetical pleotropic regions have been previously reported in petunia^27^. Such co-localization of QTL is also commonly observed in other crops such as wheat^43^, rice^44^, and brassicas^45^. The clustered QTL might also be QTL for closely linked traits from linkage disequilibrium, as the traits with co-localizing QTL were often significantly correlated (Table S3). Further discrimination from pleiotropy to linkage can be achieved by forward genetics approaches such as fine mapping or association mapping to increase the number of association events to improve the genetic resolution for these regions; or by reverse genetics approaches such as TILLING (Targeting Induced Local Lesions in Genomes) or gene specific modifications to verify the function of individual genes.

QTL mapping using both populations may help explore the extent of genetic architecture for interspecific variation. We found most QTL for the same traits were located on different chromosomes for the AE and IA populations. This is not surprising considering that *P. exserta* and *P. axillaris* diverged more recently than *P. integrifolia* and *P. axillaris* ^46^ and the populations harbor different polymorphic loci.

In plant breeding, introgression of QTL located in regions with reduced recombination might introduce unfavorable traits, which could delay the target traits introgression process. Uneven QTL distribution has been reported in other crops. For example, a comprehensive analysis integrating sorghum whole genome sequence information with 48 QTL studies found that QTL and genes were distributed unevenly across the genome, with 22% of QTL either entirely or partially located in the heterochromatic regions surrounding the centromeres, which exhibit suppressed recombination^47^. While in the IA population, the three QTL-rich regions on chromosomes 1, 2, and 5 did not seem to overlap with the regions with high SNP frequency and only represented 8–15 genomic scaffolds with 29 to 204 unigenes within 1,000 bp of the SNPs (Fig. 2; Table S10). Transcripts on the scaffolds in these regions could be surveyed for candidate genes for the identified QTL. However, since fewer RILs were phenotyped for the IA population, and the IA population had a higher percentage of residual heterozygosity than the AE population, the exact QTL locations need to be further verified. Nevertheless, the difference in the genetic location for the QTL clusters in the two populations might represent the greater genetic divergence between *P. axillaris* and *P*. *integrifolia* than between *P. exserta* and *P. axillaris*. Additionally, the QTL-rich regions in the AE population might be narrowed down by utilizing corresponding markers between the AE and IA populations, assuming the QTL for same trait were located in syntenic regions. However, the possibility of missing genomic regions/scaffolds and/or bias based on methylation and restriction site abundance from GBS-based linkage maps could miss genomic regions containing candidate genes^48^.

In summary, the high-density genetic linkage maps from two RIL populations represented an important contribution to understanding the genomic complexity of *Petunia*. QTLs detected from the present study increased our understanding of the genetics underlying plant development rate and other important crop timing and quality traits in *Petunia*. The combined results from QTL mapping and DEG identification will aid future efforts to identify and characterize novel genes involved in development rate control. Additionally, the availability of these SNP-genotyped RIL populations, the first for *Petunia*, combined with the *P. axillaris* and *P. inflata* genome sequences^7^, greatly improve the capacity for genetic mapping and gene discovery in *Petunia*.

## Materials and Methods

### Plant materials and growth conditions

The *P*. *axillaris* × *P*. *exserta* F_7_ RIL population (AE) was generated through single seed descent from the cross of *P*. *axillaris* (PI 667515) and *P*. *exserta* (OPGC943); the *P*. *integrifolia* × *P*. *axillaris* F_7_ RIL population (IA) was generated through single seed descent from the cross of *P*. *integrifolia* (purchased from Diane’s Flower Seeds, Ogdon, UT) and *P*. *axillaris* (PI 667515). A total of 171 lines from the AE population and 133 lines from the IA population were used for the subsequent phenotyping. All plants were grouped in a single experiment with three replicates per RIL and parent. Seeds of the parental species and RILs were sown and placed in a greenhouse at 23 °C. After 14 d (when seedlings had unfolded two true leaves), seedling trays for both populations were moved to a greenhouse at 20°C under a 16-h photoperiod. Five weeks after seed sow, the plants were transplanted and grown under three growing temperatures, 14°C, 17°C, and 20 °C under 16-h photoperiod.

For both populations, the number of nodes (leaves) on the primary shoot was determined on the first day plants were placed in temperature treatments, and again 14 (AE only) and 28 d later. Development rate (DRate) was then calculated as the increase in node number over a period of time (in nodes d^−1^). On the day the first flower opened, node number below first flower (Nodes), days to anthesis of the first flower (DTA), height to the first flower node (Height), diameter of first flower (FIDiam), length (LLeng) and width (LWid) of the third leaf below the flowering node were determined. Additionally, for the IA population only, the total number of visible flower buds (FIBud), the number of visible flower buds on the main stem (for plants where the first flower opened on the main stem) (FIBudPS), the number of lateral branches >8 cm in length (Branch), and the number of lateral shoots with visible flower buds (FlBranchNum) were determined on the day the first flower opened. Also, for the IA population, the first flower opened on a lateral branch instead of the primary shoot for some RILs under some temperatures^49^. In these cases, instead of Nodes, the node number from the base of the plant up to the flowering lateral shoot (NBL), the number of nodes on the flowering lateral branch up to the first open flower (NodesLB), and the leaf number on the primary shoot (LNMS) were determined. Also for these cases, the height from the soil surface to the flowering side branch (HSB) and the height of the main stem (to the apical meristem) at flowering (HMS), were determined. Quantitative genetic analysis of these traits was conducted as described in Supplementary Methods.

### Genotyping and linkage map construction

Total genomic DNA was isolated from leaf or mixed flower development stage tissues using QIAGEN DNeasy Plant Mini Kit (QIAGEN, Germantown, MD). The DNA concentration was quantified using Qubit ™dsDNA BR Assay Kit (Life Technologies, Thermo Fisher Scientific Inc.) with a Qubit™ 2.0 Fluorometer (Life Technologies, Thermo Fisher Scientific Inc.). DNA from two parental lines and 188 RILs were sent to Data2Bio, LLC (D2B; Ames, IA) for genotyping and draft linkage mapping with tunable genotyping by sequencing (tGBS; see Supplementary Methods)^50^.

Because the *P*. *axillaris* accession used for RIL population was not the same genotype as the reference genome, the genotyping data from D2B were further corrected based on the consistency of the parental genotypes and the progeny. Briefly, for loci where the genotype of the parental line *P*. *axillaris* was different than the reference *P. axillaris* genome, the genotypes of the entire population was switched to the other genotype. The initial marker orders were the genetic locations on the draft genetic linkage map. A custom Python script (Python 2.7.8)^51^ was developed to combine adjacent markers with no recombination into bins. The recombinant breakpoints were assumed to be at the boundary of adjacent bins with different genotypes. The heterozygous and missing genotypes were not considered as different genotypes. For some bins, the locations of the recombination breakpoints on the linkage map could not be determined, because of missing data between the different genotypes. Therefore, these bins were divided into sub-bins with one representative genotype in each sub-bin. These SNPs were not further grouped into a specific sub-bin due to the large amount of missing data. The representative genotypes for each bin were called for the most abundant genotypes (that were not genotyped as missing) with a custom Python script.

The genotypes from each bin were used to generate genetic linkage maps with JoinMap 4.0^52^. The regression mapping with Kosambi mapping function^53^ with the default parameters, and LOD of 15 was set to establish linkage groups. Linkage group numbers were named according to the anchor markers (SSR or CAPS) on the previous map for the IA population and the corresponding SNP markers on the bin map for the AE population. Briefly, the SNPs within each bin were placed back to the newly generated bin-map, and the transcripts where the SSR or CAPS markers were developed were mapped to the reference genome of *P*. *axillaris* with GMAP^54^. Scaffold locations for the markers on both maps were compared to determine the nearest location of SNPs to the SSR loci. For the AE map, only SNPs obtained from tGBS were used in the bin map. For the IA population, the draft genetic linkage maps yielded fragmented linkage groups. Therefore, SSR and CAPS markers from previous research^6^ were added to link fragmented linkage groups. Genotyping of SSR and CAPS followed our previous protocol^6^. SNP frequency was calculated as the percentage of total mapped SNPs at each bin interval. Syntenic relationships between the AE and IA linkage groups were detected by placing all SNP markers back to the bin map, and all SSR and CAPS markers back to the *P*. *axillaris* draft genome assembly. The markers’ physical locations on the scaffolds were compared on the corresponding linkage groups for both populations.

### QTL mapping

MapQTL 6 was used for QTL detection^55^. The genome-wide LOD significance thresholds were determined for each trait by permutation test with 1,000 permutations at an error rate of 5%. Interval mapping was performed to identify main QTL, then the strongest markers under the QTL were chosen as co-factors to fit the multiple QTL mapping model (MQM)^56^. MQM sessions were performed until detection of QTL with greater values of LOD and explained variance. The 1-LOD confidence interval was calculated for each QTL peak. QTL detection was performed for all three replicates and for the trait average. The reported QTL were from the average of three replications. QTL detected in at least two replicates within 5 cM to each other and less than 5 cM to the QTL detected using the traits average were reported as “robust QTL”. QTL detected in more than two repeats at locations >5 cM were micro-environment QTL. The robust QTL were reported using the average of the replicates. The percentage of phenotypic variation explained (PVE) by QTL (R^2^%) and their additive effect on traits at significant LOD (P ≤ 0.05) was determined. The QTL and linkage groups were visualized with Circos^57^. After QTL were detected for all traits, QTL density was calculated as number of QTL (with 1-LOD support intervals) per 1 cM along each linkage group.

### Identification of differentially expressed genes associated with development rate

Shoot apex tissues were collected from four “fast-developing” (IA138, IA160, IA288, and IA349) and four “slow-developing” (IA161, IA41, IA474, and IA476) IA RILs, as determined by the phenotyping described above, growing in a common environment (17 °C and a photoperiod of 9 h) 11 weeks after planting, and replicated in two different greenhouse compartments. Total RNA was extracted using an RNeasy Plant Mini Kit (Qiagen, Valencia, CA). An additional purification was performed using mini spin columns (Qiagen, Valencia, CA). The RNA qualification and quantification was determined using The Caliper Labchip GX system (Perkin Elmer, Walthan, Massachusetts, USA) at the Michigan State University Research Technology Support Facility (RTSF; http://rtsf.msu.edu/; East Lansing, MI). The sequencing libraries were prepared using the Illumina TruSeq Stranded mRNA LT Kit. Eight libraries from each greenhouse were pooled for multiplexed sequencing on one lane of an Illumina HiSeq 2500 Rapid Run flow cell (v1) at the Michigan State University Research Technology Support Facility (RTSF; http://rtsf.msu.edu/; East Lansing, MI). Sequencing was performed in a 1×50 bp format using Rapid SBS Reagents. Online base calling was done by Illumina Real Time Analysis (RTA) v1.18.61 and output of RTA was demultiplexed and converted to FastQ by Illumina Bcl2fastq v1.8.4 (Ilumina Inc. San Diego, CA, USA). Raw read quality was assessed with FastQC (http://www.bioinformatics.babraham.ac.uk/projects/fastqc/). Sequences were filtered and trimmed based on quality metrics and adapter sequences were removed with TrimmomaticSE^58^ (version 0.30). The TrimmomaticSE options employed included SLIDINGWINDOW5:20 MINLEN: 30, and HEADCROP: 14. Cleaned reads were reassessed with FastQC for quality visualization to ensure no further filtering was required. The filtered reads were aligned to the *P*. *axillaris* reference transcriptome^6^ with TopHat2 (version 2.0.8b)^59^. Read counts were used to find differentially expressed transcripts with the R package DESeq2 (version 1.4.5)^60^. Differentially expressed transcripts with an adjusted P less than 0.05 found between the fast and the slow RIL groups were considered as differentially expressed. The annotation of these transcripts were from a previous report^6^. Annotated unigenes containing SNPs were visualized by BGI WEGO (web gene ontology annotation plotting) (http://wego.genomics.org.cn/cgi-bin/wego/index.pl).

## Electronic supplementary material

Supplementary information

Table S3

Table S4

Table S5

Table S6

Table S7

Table S11

Table S12
