# Supplementary material for: Genetic Determinants of Crop Timing and Quality Traits in Two Interspecific Petunia Recombinant Inbred Line Populations
Source: Sci Rep. 2017 Jun 9;7:3200. doi: 10.1038/s41598-017-03528-9 (PMC5466624; doi:10.1038/s41598-017-03528-9)
Supplement: Supplementary file 1 — Supplementary information [file 41598_2017_3528_MOESM1_ESM.pdf]

Genetic Determinants of Crop Timing and Quality Traits in Two Interspecific Petunia Recombinant Inbred Line populations

Yufang Guo, Wei-Kuang Lin, QiuXia Chen, Veronica A. Vallejo and Ryan M. Warner[1]

Department of Horticulture, Michigan State University, East Lansing, MI 48824

[1] Corresponding author: warnerry@msu.edu

Supplementary Information

Supplementary Methods

Table S1. Descriptive statistics for traits analyzed in the *P. axillaris* × *P. exserta* (AE) RIL population and their parents.

Table S2. Descriptive statistics for traits analyzed in the *P. integrifolia* × *P. axillaris* (IA) RIL population and their parents.

Table S3. Coefficient of correlation among traits for the *P. axillaris* × *P. exserta* (AE) RIL population.

Table S4. Coefficient of correlation among traits for the *P. integrifolia* × *P. axillaris* (IA) RIL population.

Table S5. Bin-based genetic linkage maps for the AE and IA populations.

Table S6. List of SNPs within each bin for the AE and IA populations, with SNPs presented by *Petunia axillaris* genome scaffold name and the physical location on scaffold.

Table S7. Corresponding bin markers between the AE and IA linkage maps. The corresponding bins were calculated based on SNPs from the two linkage maps within a bin at the same physical location, within 100 bp, or within 30,000 bp on the same scaffold.

Table S8. Summary of QTL identified for crop timing and quality traits for two interspecific *Petunia* RIL populations, *P. axillaris* × *P. exserta* (AE) and *P. integrifolia* × *P. axillaris* (IA).

Table S9. Summary of QTL for traits unique to the *P. integrifolia* × *P. axillaris* (IA) population.

Table S10. Summary of QTL-rich regions in each population.

Table S11. Comparison of corresponding QTL across the two populations. For each hypothetical pair of corresponding (same) QTL, the location on one population was projected to the other population based on the SNPs within the QTL.

Table S12. Differentially expressed transcripts between shoot apex tissues of fast- and slow-developing IA RILs. The genetic location of DEGs on the IA linkage map was determined by identifying a mapped SNP within 1,000,000 bp of the DEG on the *P. axillaris* genome sequence.

Figure S1. Heat map generated from top 50 differentially expressed genes from apical meristem transcriptome data from the fast and slow lines in the IA RIL population.

Supplementary Methods

Quantitative genetic analyses

For each trait, total phenotypic variance was partitioned into sources attributable to genotype (V_G) and error (V_E) according to Keurentjes et al. [1]. Briefly, the random-effects model y=μ+G+E was used. The broad sense heritability (H^2) for the RIL population was estimated according to the formula $H^2=\delta^2_g/(\delta^2_g+\delta^2_e)$, where δ^2_g (V_G) is the between genotype variance component and δ^2_e (V_E) is the residual variance. The coefficient of genetic variation CV_g was estimated as $CV_g = (100\times \sqrt{VG})/\bar{X}$, where $\bar{X}$ is the trait mean. Analysis of variance (for the RIL population), Pearson correlation coefficients (for traits correlation), and independent samples t-test (to compare parental means) were calculated by using IBM SPSS Statistics 22 (Armonk, NY: IBM Corp).

Genotyping and linkage map construction

Sequencing was performed at four HiSeq Single-End Lanes on a Genome Analyzer 2000 (2000, Inc, San Diego, CA). The reference genome was from a *P. axillaris* genotype 1.26 Gb in size and consists of 83,639 scaffolds[2]. The quality trimmed reads were mapped to the reference genome using GSNAP[3]. Only unique alignments were used for SNP calling. Both homozygous and heterozygous SNPs were called with PHRED quality ≥20 (≤1% error rate) with at least five reads under the SNP loci; while the first and last three base pairs of each read were ignored. The SNPs with an allele frequency ≥0.8 were called as homozygous. Heterozygous SNPs were called when each allele had an allele frequency ≥0.3, and the overall allele frequency was ≥0.8. The SNPs were transformed into genotyping data based on the following criteria: for the homozygous call, major alleles had at least five reads with allele frequency of ≥0.9, for the

heterozygous call, each allele had at least two reads with allele frequency ≥0.2, and the sum of the two alleles had at least five reads and a combined allele frequency of ≥0.9. The putative SNPs were then filtered with the following criteria: missing data rate ≤ 80%, each locus had only two alleles and at least two genotypes with minor allele frequency (MAF) ≥0.1. With the filtered SNPs, individual RIL were removed based on the percentage of missing data (≥90% of SNPs with no genotype calls) and the heterozygous rate (heterozygous SNP site >25% for the AE population and >30% for the IA population). Subsequent SNP filtering was performed to filter samples in the RIL population for overall missing data rate ≤50%, and with only two alleles for at least two genotypes, the samples should have heterozygosity limited to 0 to 10% and MAF≥0.1. The filtered SNPs and RILs were used for a draft genetic linkage map by R/qtl [4]. For the AE population, the parameters for linkage mapping were: maximum estimated recombination fraction (rf) of 0.25, LOD (logarithm of odds) score ≥16, and at least 100 SNPs per linkage group. A higher percentage of heterozygosity existed for the IA population, therefore, the parameters for IA population linkage map construction were: maximum estimated recombination fraction (rf) of 0.1, LOD score ≥ 30 and at least 20 SNPs per linkage group. The more relaxed parameters for the IA population enabled more markers to be placed onto the map and minimized the number of linkage groups.

1 Keurentjes, J. J. *et al.* Development of a near-isogenic line population of Arabidopsis thaliana and comparison of mapping power with a recombinant inbred line population. *Genetics* **175**, 891-905, doi:10.1534/genetics.106.066423 (2007).
2 Bombarely, A. *et al.* Insight into the evolution of the Solanaceae from the parental genomes of Petunia hybrida. *Nature Plants* **Article Number: 16074** (2016).
3 Wu, T. & Nacu, S. Fast and SNP-tolerant detection of complex variants and splicing in short reads. *Bioinformatics* **26**, 873-881 (2010).
4 Broman, K., Wu, H., Sen, Ś. & Churchill, G. R/qtl: QTL mapping in experimental crosses. *Bioinformatics* **19**, 889-890 (2003).

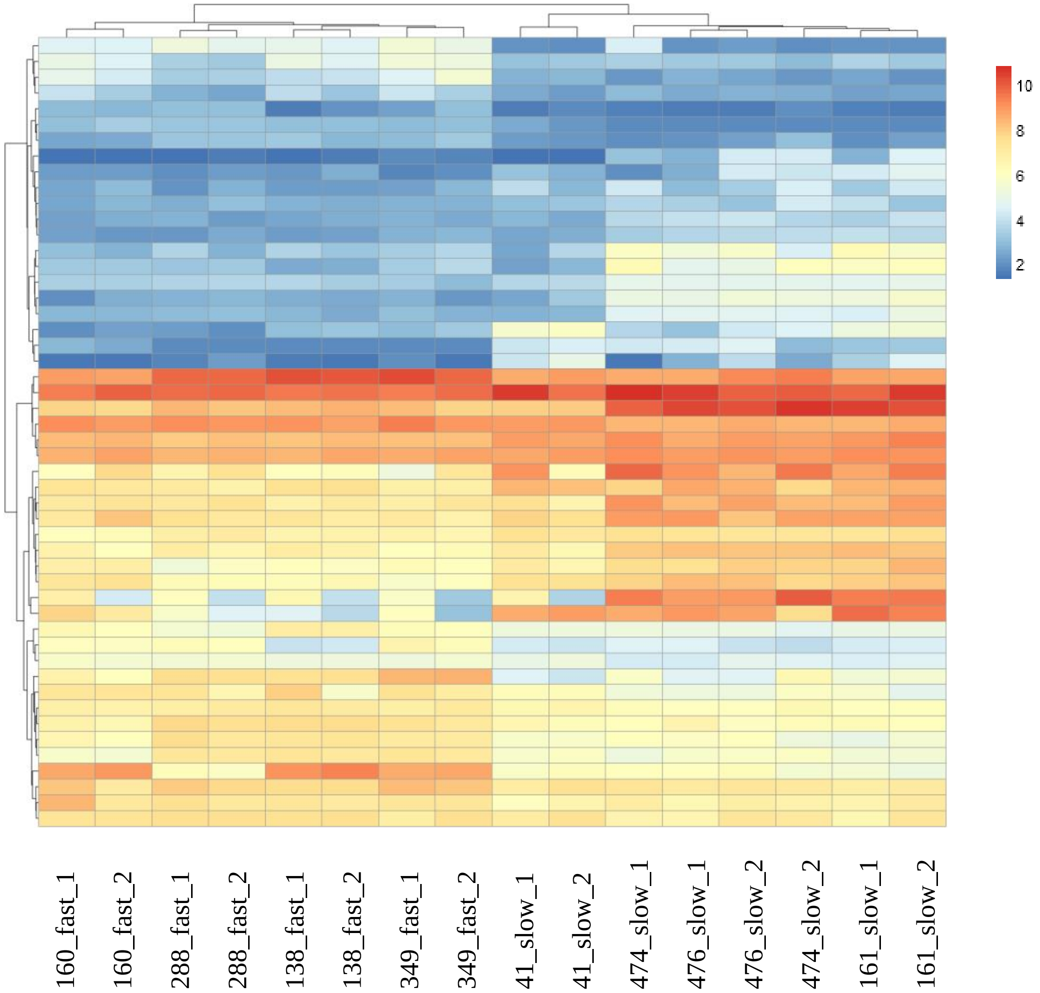

Supplementary Figure S1. Heat map generated from top 50 differentially expressed genes from apical meristem transcriptome data from the fast and slow lines in the IA RIL population.

Supplementary Table S1. Descriptive statistics for traits analyzed in the *P. axillaris*× *P. exserta* (AE) RIL population and their parents.

Trait	Parent	Temp	$\bar{x}$±(SD)	Range	$\bar{x}$±(SD)	Range	[V_G]	[V_E]	[H^2]	[CV_G]	Skewness	Kurtosis
			Parents		RIL population							
Drate (nodes day^{-1})	PA (Interval_1_14)	14	0.38±0.08	0.29-0.43	0.31±0.07	0.07-0.50	0.002	0.003	0.4	14.43	-0.40	1.30
	PE (Interval_1_14)	14	0.36±0.07	0.29-0.43								
Drate (nodes day^{-1})	PA (Interval_14_28)	14	0.48±0.08	0.43-0.57	0.38±0.07	0.21-0.57	0.001	0.004	0.2	8.32	0.40	0.40
	PE (Interval_14_28)	.4	0.33±0.08	0.29-0.43								
Drate (nodes day^{-1})	PA (Interval_1_28)	.4	0.43±0.07	0.36-0.50	0.34±0.06	0.14-0.54	0.001	0.002	0.33	9.30	-0.10	1.00
	PE (Interval_1_28)	.4	0.35±0.07	0.29-0.43								
Nodes	PA	.4	28.33±1.53*	27.00-30.00	17.64±2.39	11.00-27.00	4.57	1.16	0.80	12.12	0.78	0.22
	PE	.4	16.33±0.58	16.00-17.00								
DTA	PA	.4	70.00±4.58*	65.00-74.00	62.68±8.11	46.00-93.00	18.42	47.38	0.28	6.85	0.89	1.20
	PE	.4	56.33±6.43	49.00-61.00								
HghtFI (mm)	PA	.4	218.30±36.20	195.00-260.00	213.80±2.8	70.00-490.00	2650.00	130.8.0	0.67	24.080	0.4.80	0.350
	PE	.4	170.00±10.00	160.00-180.00								
FIDiam (mm)	PA	.4	55.00±0.00**	55.00-55.00	53.80±6.50	35.00-75.00	22.00	20.00	0.52	8.72	0.10	-0.29
	PE	.4	50.00±0.00	50.00-50.00								
LLeng (mm)	PA	.4	56.70±10.40	45.00-65.00	106.80±20.70	60.00-1410.00	218.30	210.50	0.51	13.83	0.42	0.24
	PE	.4	113.30±15.30	100.00-130.00								
LWid (mm)	PA	.4	28.30±7.60*	20.00-35.00	50.00±10.80	15.00-90.00	63.00	53.00	0.54	15.88	0.3	0.08
	PE	.4	60.00±5.00	55.00-65.00								
Drate	PA (Interval_1_14)	.7	0.48±0.04	0.43-0.50	0.40±0.06	0.21-0.79	0.001	0.003	0.25	7.91	0.75	3.12
	PE (Interval_1_14)	.7	0.45±0.04	0.43-0.50								
DRate	PA (Interval_14_28)	.7	0.64±0.07	0.57-0.71	0.54±0.08	0.14-0.79	0.003	0.003	0.5	10.14	-0.16	1.84
	PE (Interval_14_28)	.7	0.50±0.00	0.50-0.50								
DRate	PA (Interval_1_28)	.7	0.56±0.05	0.50-0.61	0.47±0.06	0.25-0.64	0.002	0.002	0.5	9.52	0.15	0.78

	PE (Interval_1_28)	.7	0.48±0.02	0.46-0.50								
Nodes	PA	.7	26.00±1.73*	24.00-27.00	17.16±2.34	12.00-28.00	4.25	1.24	0.77	12.01	1.10	2.63
	PE	.7	15.67±0.58	15.00-16.00								
DTA	PA	.7	51.00±3.61*	47.00-54.00	44.49±4.24	37.00-70.00	8.67	9.30	0.48	6.62	1.19	3.46
	PE	l7	41.33±2.52	39.00-44.00								
HghtFI (mm)	PA	.7	280.00±28.40*	250.00-305.00	213.30±57.50	70.00-380.00	2381.00	932.00	0.72	22.88	0.25	-0.21
	PE	17	171.70±2.90	170.00-175.00								
FIDiam (mm)	PA	17	53.30±2.90	50.00-55.00	50.90±6.60	30.00-75.00	21	23	0.48	9	0.06	0.28
	PE	l7	48.30±2.90	45.00-50.00								
LLeng (mm)	PA	.7	85.00±18.00	70.00-105.00	113.60±20.10	40.00-175.00	206	200	0.51	12.63	0.25	0.11
	PE	.7	123.30±2.90	120.00-125.00								
LWid (mm)	PA	.7	38.30±5.80*	35.00-45.00	54.50±11.60	15.00-150.00	63	73	0.46	14.50	1.41	9.62
	PE	.7	66.70±2.90	65.00-70.00								
Drate	PA (Interval_1_14)	20	0.52±0.08	0.43-0.57	0.43±0.06	0.07-0.64	0.001	0.003	0.25	7.35	-0.56	2.66
	PE (Interval_1_14)	20	0.48±0.08	0.43-0.57								
Drate	PA (Interval_14_28)	20	0.71±0.07	0.64-0.79	0.61±0.10	0.14-1.00	0.005	0.005	0.5	11.59	0.16	1.24
	PE (Interval_14_28)	20	0.52±0.04	0.50-0.57								
Drate	PA (Interval_1_28)	20	0.62±0.07	0.54-0.68	0.52±0.06	0.25-0.75	0.002	0.002	0.5	8.60	0.10	1.96
	PE (Interval_1_28)	20	0.50±0.04	0.46-0.54								
Nodes	PA	20	25.00±1.03	25.00-26.00	17.33±2.62	13.00-30.00	5.38	1.48	0.78	13.38	1.08	2.29
	PE	20	15.67±1.16	15.00-17.00								
DTA	PA	20	45.33±1.53**	44.00-47.00	39.55±4.40	31.00-62.00	11.72	7.67	0.60	8.66	0.93	1.37
	PE	20	35.33±1.53	34.00-37.00								
HghtFI (mm)	PA	20	243.30±20.80*	220.00-260.00	207.20±59.00	35.00-390.00	2425.00	1068.00	0.69	23.77	0.43	0.06
	PE	20	125.00±5.00	120.00-130.00								
FIDiam (mm)	PA	20	50.00±5.00	45.00-55.00	48.20±6.60	35.00-70.00	20.00	23	0.47	9.28	0.49	0.16
	PE	20	40.00±0.00	40.00-40.00								
LLeng (mm)	PA	20	83.30±24.70	55.00-100.00	119.40±23.10	35.00-195.00	270.00	264	0.51	13.76	0.12	0.24

	PE	20	130.00±10.00	110.00-130.00								
LWid (mm)	PA	20	33.30±11.60	20.00-40.00	56.90±12.40	20.00-95.00	91.00	62.00	0.59	16.77	0.32	0.01
	PE	20	65.00±5.80	55.00-65.00								

*Significant between parents at 95% confidence level

**The standard error of difference is 0, t test cannot be computed

***restricted maximum likelihood

Supplementary Table S2. Descriptive statistics for traits analyzed in the *P. integrifolia* × *P. axillaris* (IA) RIL population and their parents.

Trait	Parent	Temp (ºC)	$\bar{x}$±(SD)	Range	$\bar{x}$±(SD)	Range	[V_G]	[V_E]	[H^2]	[CV_G]	Skewness	Kurtosis
			Parents		RIL population							
Drate (nodes day^{-1})	PA	14	0.36±0.04*	0.32-0.39	0.31±0.08	0.04-0.57	0.003	0.003	0.5	17.67	-0.3	1.05
	PI	14	0.25	0.25-0.25								
DTA	PA	14	81.33±5.03*	76.00-86.00	94.86±10.37	76.00-140.00	72.52	35.55	0.67	8.98	1.18	2.51
	PI	14	101.00±2.83	99.00-103.00								
Nodes	PA	14	27.00±3.61	23.00-30.00	24.03±4.57	10.00-36.00	10.15	10.84	0.48	13.26	-0.27	1.00
	PI	14	-	-								
NBL	PA	14	-	-	3.45±1.51	1.00-11.00	-0.11	2.38	***	***	1.27	3.91
	PI	14	1.00	1.00-1.00								
NodesLB	PA	14	-	-	17.71±4.76	9.00-40.00	15.00	7.81	0.66	21.87	1.37	2.94
	PI	14	14.5±3.54	12.00-17.00								
LNMS	PA	14	-	-	28.45±5.74	13.00-43.00	11.05	21.96	0.33	11.68	0.30	0.48
	PI	14	23.00±2.83	21.00-25.00								
Branch	PA	14	12.67±0.58	12.00-13.00	12.18±3.52	1.00-25.00	6.71	5.69	0.54	21.27	0.58	2.57
	PI	14	8.00±1.41	7.00-9.00								
Height (mm)	PA	14	254.00±34.51	220.00-289.00	237.29.09±69.59	72.00-450.00	2934.61	1947.74	0.47	22.83	0.13	0.10
	PI	14	-	-								
HMS (mm)	PA	14	-	-	143.56±67.70	22.00-380.00	3263.15	1349.58	0.60	39.79	0.86	0.86
	PI	14	108.5±50.2	73.00-144.00								
HSB (mm)	PA	14	-	-	156.07±71.35	8.00-350.00	2319.61	2793.04	0.45	30.86	0.52	-0.06
	PI	14	129.50±44.55	98.00-161.00								
FDiam (mm)	PA	14	57.33±28.15	31.00-87.00	49.91±9.49	19.00-73.00	54.25	36.23	0.60	14.76	0.05	-0.28
	PI	14	50.00±11.31	42.00-58.00								
FIBudPS	PA	14	14.00±5.57	9.00_20.00	16.51±8.48	1.00-50.00	26.98	45.02	0.37	31.46	0.72	0.43

	PI	14	12.00±1.41	11.00-13.00								
FIBud	PA	14	90.67±28.89	73.00-124.00	139.38±77.03	10.00-454.00	3119.69	2833.93	0.52	40.07	1.31	2.32
	PI	14	86.00±39.60	58.00-114.00								
FIBranchNum	PA	14	12.67±0.58*	12.00-13.00	12.13±3.34	1.00-23.00	6.23	4.95	0.56	20.58	-0.04	0.82
	PI	14	9.00	9.00								
LLeng (mm)	PA	14	70.00±8.00	29.00-38.00	50.74±14.84	8.00-118.00	92.33	128.4	0.42	18.94	0.96	2.35
	PI	14	48.00±12.73	39.00-57.00								
Lwid (mm)	PA	14	33.33±4.51	29.00-38.00	24.99±8.44	4.00-61.00	33.38	38.07	0.47	23.12	0.86	1.39
	PI	14	30.50±12.02	22.00-39.00								
Drate (nodes day^{-1})	PA	17	0.45±0.05	0.39-0.50	0.37±0.09	0.07-0.68	0.004	0.005	0.44	17.09	0.14	1.03
	PI	17	0.32±0.06	0.25-0.36								
DTA	PA	17	70.00±2.65*	68.00-73.00	79.63±9.43	64.00-139.00	48.3	40.92	0.54	8.73	2	7.79
	PI	17	91.33±1.15	90.00-92.00								
Nodes	PA	17	24.00±1.00	23.00-25.00	24.08±4.46	10.00-41.00	9.17	10.75	0.46	12.58	0.54	1.81
	PI	17	23.00±0.00	23.00-23.00								
NBL	PA	17	-	-	3.65±1.55	1.00-10.00	1.54	0.89	0.63	34.00	1.18	3.83
	PI	17	1.00	1.00-1.00								
NoedsLB	PA	17	-	-	15.45±3.30	9.00-23.00	5.23	5.77	0.48	14.80	0.11	-0.56
	PI	17	19.00	19.00-19.00								
LNMS	PA	17	-	-	26.94±4.89	13.00-40.00	7.03	16.96	0.29	9.84	-0.06	0.34
	PI	17	14.00	14.00-14.00								
Branch	PA	17	11.67±1.53*	10.00-13.00	11.25±3.20	1.00-22.00	5.18	5.12	0.50	20.23	-0.21	1.40
	PI	17	8.00±1.00	7.00-9.00								
Height (mm)	PA	17	247.67±33.86	217.00-284.00	249.85±80.86	58.00-452.00	3316.40	3246.75	0.51	23.05	-0.02	-0.54
	PI	17	158.50±41.72	129.00-188.00								
HMS (mm)	PA	17	-	-	133.17±57.74	20.00-270.00	2118.46	1248.96	0.63	34.56	0.43	-0.20
	PI	17	134.00	134.00-134.00								
HSB	PA	17	-	-	134.77±45.40	39.00-273.00	787.12	1287.26	0.38	20.82	0.67	0.48

(mm)											
	PI	17	256.00	256.00-256.00							
FDiam (mm)	PA	17	56.00±2.65	53.00-58.00	49.40±8.98	11.00-74.00	44.13	36.88	0.54	13.45	-0.26	1.14
	PI	17	52.67±6.66	47.00-60.00							
FIBudPS	PA	17	14.33±5.03	9.00-19.00	19.87±9.45	3.00-91.00	39.70	49.85	0.44	31.71	2.12	13.00
	PI	17	23.67±6.51	17.00-30.00							
FIBud	PA	17	76.33±30.27	44.00-104.00	121.96±57.07	4.00-322.00	1626.45	1641.76	0.50	33.07	0.74	0.84
	PI	17	136.33±35.02	96.00-159.00							
FIBranchNum	PA	17	11.67±1.53*	10.00-13.00	11.33±3.19	1.00-22.00	4.92	5.29	0.48	19.58	-0.18	1.42
	PI	17	8.00±1.00	7.00_9.00							
LLeng (mm)	PA	17	66.00±12.29	52.00-75.00	57.98±17.42	18.00-158.00	107.71	196.31	0.35	17.90	1.57	5.53
	PI	17	44.33±7.02	37.00-51.00							
LWid (mm)	PA	17	34.00±7.00	26.00-39.00	28.63±9.92	5.00-74.00	56.52	42.25	0.57	26.26	1.06	2.10
	PI	17	26.67±2.31	24.00-28.00							
Drate (nodes day^{-1})	PA	20	0.48±0.03	0.46-0.50	0.50±0.11	0.14-0.89	0.006	0.005	0.55	15.49	0.24	0.85
	PI	20	0.44±0.05	0.39-0.50							
DTA	PA	20	68.00±4.24*	65.00-71.00	68.56±8.11	36.00-128.00	29.95	35.94	0.45	7.98	1.77	11.89
	PI	20	86.67±3.06	84.00-90.00							
Nodes	PA (not comparable)	20	29.00**	29.00	24.83±4.19	10.00-38.00	9.26	8.34	0.52	12.26	0.13	0.54
	PI	20	25.00(only one plant)	only one plant							
NBL	PA	20	-	-	2.96±1.00	1.00-7.00	0.44	0.57	0.44	22.41	1.41	3.89
	PI	20	3.00±1.41	2.00-4.00							
NodesLB	PA	20	-	-	14.75±3.71	10.00-30.00	7.31	6.61	0.53	18.33	1.48	3.87
	PI	20	15.00±2.83	13.00-17.00							
LNMS	PA	20	-	-	26.82±3.86	20.00-35.00	6.41	8.59	0.43	9.44	0.13	-0.56
	PI	20	23.50±2.13	22.00-25.00							
Branch	PA	20	8.50±0.71*	8.00-9.00	8.52±2.67	1.00-27.00	3.46	3.71	0.48	21.83	1.29	7.70

	PI	20	6.33±0.58	6.00-7.00								
Height (mm)	PA	20	206.50±146.37 **	103.00-310.00	266.64±90.97	50.00-520.00	5598.72	2714.50	0.67	28.06	0.10	-0.30
	PI	20	195.00(only one plant)	only one plant								
HMS (mm)	PA	20	-	-	172.81±68.10	28.00-392.00	2912.38	1789.5	0.62	31.23	0.50	0.99
	PI	20	191.00±57.98	150.00-232.00								
HSB (mm)	PA	20	-	-	184.14±66.86	90.00-340.00	2784.68	1746.61	0.61	28.66	0.56	-0.58
	PI	20	208.50±33.23	185.00-232.00								
FDiam (mm)	PA	20	58.00±8.49	52.00-64.00	47.29±7.85	26.00-73.00	39.84	21.94	0.64	13.35	0.38	0.18
	PI	20	48.67±1.15	48.00-50.00								
FIBudPS	PA	20	21.50±14.85	11.00-32.00	16.42±7.00	2.00-39.00	22.75	26.37	0.46	29.05	0.49	0.07
	PI	20	18.67±10.79	11.00-31.00								
FIBud	PA	20	81.00±31.11	59.00-103.00	78.28±44.01	3.00-282.00	1286.29	657.98	0.66	45.82	1.73	4.76
	PI	20	75.00±22.52	49.00-88.00								
FIBranchNum	PA	20	8.50±0.71*	8.00-9.00	8.48±2.62	1.00-22.00	3.56	3.32	0.52	22.25	0.50	2.47
	PI	20	6.33±0.58	6.00-7.00								
LLeng (mm)	PA	20	55.50±7.78	50.00-61.00	58.45±15.98	10.00-111.00	119.11	136.85	0.47	18.67	0.45	0.33
	PI	20	56.67±3.51	53.00-60.00								
LWid (mm)	PA	20	28.00(only one plant)	only one plant	29.23±9.71	6.00-61.00	61.03	33.65	0.64	26.73	0.63	0.32
	PI	20	33.00±2.64	30.00-35.00								

*Significant between parents at 95% confidence level

**The standard error of difference is 0, t test cannot be computed

***restricted maximum likelihood

Table S8. Summary of QTL identified for crop timing and quality traits for two interspecific *Petunia* RIL populations, *P. axillaris* × *P. exserta* (AE) and *P. integrifolia* × *P. axillaris* (IA).

Trait	LOD threshold	QTL	Chr.	Position (cM)	Nearest Marker	Support interval (1 LOD)	LOD	Explained variance (%)	Additive effects	Trait	LOD threshold	QTL	Chr.	Position (cM)	Nearest Marker	Support interval (1 LOD)	LOD	Explained variance (%)	Additive effects
**AE**										**IA**									
Nodes_14	2.7	14NBF2.1	2	22.544	AE_bin56_26_229_2	22.514-22.544	7.63	10.3	0.768										
		14NBF3.1	3	4.317	AE_bin104_1	3.45-5.317	4.54	5.8	0.573										
		14NBF4.1*	4	24.045	AE_bin210_117_218_4_1_2	25.997-26.045	22.23	37.7	1.435										
		14NBF6.1*	6	34.826	AE_bin272_3_88_2	34.704-34.826	9.84	13.7	0.941										
Nodes_17	2.6	17NBF2.1	2	7.496	AE_bin12-2	7.111-7.496	4.07	5.9	0.558	Nodes_17	2.7	17NBF2.1	2	21.441	IA_bin154_2	21.107-21.441	2.84	8.9	-1.76
		17NBF3.1	3	11.884	AE_bin106_7	7.884-19.276	4.69	6.6	0.579			17NBF2.2	2	33.796	IA_bin180_5	33.575-33.796	5.31	17.9	2.42
		17NBF4.1*	4	27.839	AE_bin206_4	27.494-27.839	18.11	32.6	1.272			17NBF1.1	5	5.894	IA_bin123_2	5.521-5.894	4.8	16	1.92
		17NBF6.1*	6	33.163	AE_bin275_3_88_1	33.035-33.163	4.22	6.1	0.572										
Nodes_20	2.4	20NBF2.1*	2	36.409	AE_bin63_260_253_2	36.409-36.599	5.08	7.6	0.9555	Nodes_20	2.7	20NBF5.1	5	11.04*	IA_bin143_4	11.651-11.040	9.22	34.3	2.45
		20NBF3.1	3	14.276	AE_bin105_7	8.884-20.276	3.49	5.1	0.574										
		20NBF4.1*	4	27.839	AE_bin206_4	27.494-27.839	15.70	27.6	1.326										
		20NBF6.1	6	33.948	AE_bin258_6_214_1	33.658-33.948	4.39	6.5	0.676										
DTA_14	2.7	14DTA2.1*	2	22.544	AE_bin56_26_229_2	22.514-22.544	13.73	31.2	3.506	DTA_14	2.8	14DTA1.1	1	34.171	IA_bin100_5	33.44-34.171	6.04	24.1	6.21
		14DTA4.1	4	27.839	AE_bin206_4	27.494-27.839	3.13	6.1	1.497										
DTA_17	2.6	17DTA2.1*	2	22.544	AE_bin56_26_229_2	22.514-22.544	11.49	21.2	1.667	DTA_17	2.7	17DTA1.1*	1	29.16	IA_bin82_1	29.106-29.160	5.83	19.5	5.24
		17DTA3.1	3	53.127	AE_bin132_1	52.155-53.127	4.32	7.1	0.963			17DTA6.1	6	19.668	IA_bin329_1	19.376-19.668	3.92	12.5	3.61
		17DTA4.1*	4	27.839	AE_bin206_4	27.494-27.839	7.89	13.8	1.31										
DTA_20	2.8	20DTA2.1*	2	22.544	AE_bin56_26_229_2	22.514-22.544	11.25	21.8	1.86	DTA_20	2.8	20DTA1.1*	1	36.436	IA_bin105_5	36.2-36.436	5.66	20.4	3.66
		20DTA3.1	3	76.104	AE_bin164_64	76.026-76.104	2.96	5.1	0.87										
		20DTA4.1*	4	27.839	AE_bin206_4	27.494-27.839	8.04	14.8	1.51										
FlDiam_14	2.7	14FD2.1*	2	23.01	AE_bin49_8	22.995-23.010	11.91	23.3	-2.69	FlDiam_14	2.7	14FD2.1*	2	31.783	IA_bin176_3	31.589-31.783	11.38	40.5	-5.32
		14FD3.1	3	75.3	AE_bin179_69	75.254-75.3	5.1	9.2	1.59										
		14FD6.1	6	33.233	AE_bin274_33	33.163-33.233	3.89	6.9	-1.46										

		14FD7.1	7	19.722	AE_bin319_1	12.479-20.722	3.15	5.4	1.28										
FIDiam_17	2.6	17FD2.1	2	23.01	AE_bin49_8	23.010-23.289	3.48	5.5	-1.31	FIDiam_17	2.6	17FD2.1*	2	31.055	IA_bin170_3	30.8-31.055	6.08	18.7	-3.76
		17FD3.1*	3	75.254	AE_bin178_112	75.143-75.254	8.39	14.3	2.05			17FD5.1	5	5.085	IA_bin121_9	4.651-5.085	2.7	7.7	-2.55
		17FD4.1	4	26.142	AE_bin222_330_313_2	26.045-26.142	3.80	6.0	1.35										
		17FD6.1	6	32.398	AE_259_236_46_42_97_122	32.195-32.398	4.81	7.8	-1.62										
		17FD7.1*	7	21.827	AE_bin316_1	21.563-21.827	5.75	9.4	1.7										
FIDiam_20	2.6	20FD3.1*	3	75.143	AE_bin181_35	75.008-75.143	7.27	14	1.99	FIDiam_20	2.7	20FD1.1	1	11.298	IA_bin406_10_164_1	10.867-11.298	3.45	6.5	1.97
		20FD4.1	4	19.675	AE_bin200_2	19.289-20.675	4.18	7.7	1.54			20FD2.1*	2	30.77	IA_bin171_2	30.487-30.77	19.93	53.8	-5.42
		20FD6.1*	6	32.544	AE_bin259_236_46_42_97_1	32.462-32.544	5.03	9.3	-1.78										
LLeng_14	2.7	14LL3.1*	3	74.661	AE_bin159_15	74.482-74.661	7.63	15.5	-6.84	LLeng_14	2.7	14LL5.1	5	4.063	IA_bin119_24	4.063-4.348	4.51	17.2	-23.73
		14LL4.1*	4	25.573	AE_bin221_1	25.451-25.573	4.93	9.6	-5.46			14LL5.2	5	4.651	IA_bin120_2	4.348-4.561	5.46	18.9	25.79
		14LL6.1*	6	35.963	AE_bin251_3	35.755-35.963	5.66	11.1	-6.11										
LLeng_17	2.6	17LL2.1	2	31.99	AE_bin4_1	30.381-32.990	3.78	9.1	7.63	LLeng_17	2.7	17LL1.1	1	7.996	4177	7-8.996	2.76	12	5.77
		17LL3.1*	3	76.781	AE_bin171_14_39_1	76.681-76.781	4.66	11.4	-5.92										
LLeng_20	2.6	20LL2.1*	2	20.981	AE_bin44_4	20.854-20.981	6.73	12	6.81	LLeng_20	2.8	20LL6.1*	6	24.024	IA_bin358_1	23.899-24.024	4.16	15.5	6.15
		20LL3.1	3	76.557	AE_bin166_17	76.509-76.557	5.81	10.2	-6.21										
		20LL6.1	6	36.335	AE_bin252_5	35.994-36.335	3.75	6.4	-5.22										
		20LL7.1*	7	32.179	AE_bin327_49_49_1	31.994-32.179	5.24	9.1	5.94										
LWid_14	2.6	14LW3.1*	3	75.955	AE_bin174_55_296_2	75.88-75.955	14.8	35	-5.37	LWid_14	2.9	14LW1.1	1	10.867	IA_bin407_8	10.49-10.867	5.56	14.2	10.46
												14LW1.2	1	11.46	IA_bin406_10_164_1	11.298-11.46	3.63	8.8	-8.54
												14LW2.1	2	39.872	IA_bin191_9	39.039-39.872	3.55	8.6	-2.25
												14LW5.1	5	4.348	IA_bin119_24	3.063-4.348	3.36	8.1	-2.26
LWid_17	2.6	17LW2.1	2	33.912	AE_bin2_2	31.990-33.921	4.25	9.5	3.62	LWid_17	2.7	17LW1.1*	1	10.49	IA_bin403_1	10.05-10.49	5.85	16.4	4.12
		17LW3.1*	3	76.781	AE_bin171_14_39_1	76.618-76.781	7.83	18.5	-4.08			17LW2.1*	2	30.487	IA_bin172_1	30.13-30.487	5.01	13.8	-3.54
												17LW5.1	5	6.654	IA_bin125_7	5.894-6.654	3.11	8.2	-2.82
LWid_20	2.7	20LW1.1*	1	13.115	AE_bin94_2	12.699-13.115	4.98	8	-3.11	LWid_20	2.8	20LW1.1	1	11.753	IA_bin410_4	11.46-11.753	3.03	7.9	2.92
		20LW2.1*	2	22.113	AE_bin52_48	21.798-22.113	8.62	14.7	4.25			20LW2.1*	2	47.344	IA_bin224_62_288_352_22	47.291-47.344	6.96	19.7	-3.89
		20LW3.1*	3	73.553	AE_bin169_1	73.328-73.553	7.06	11.7	-3.77			20LW7.1*	7	16.26	31450	15.931-16.26	3.39	8.9	3.59

		20LW6.1	6	36.335	AE_bin252_5	35.994-36.335	2.79	4.4	-2.41										
Drate_14_1	2.7	14.1DR3.1	3	4.317	AE_bin104_1	3.45-5.317	5.36	12.9	0.021										
		14.1DR6.1	6	37.295	AE_bin269_1	37.141-38.295	3.47	8.1	0.017										
		14.1DR7.1	7	1.739	AE_bin311_1	1.02-1.739	2.91	6.8	-0.018										
Drate_14_2	2.7	14.2DR3.1	3	4.317	AE_bin104_1	3.45-5.317	5.51	13.5	0.016										
		14.2DR6.1	6	33.163	AE_bin274_33	33.163-33.233	5.18	12.6	0.016										
Drate_14_all	2.6	14.AllDR3.1*	3	4.317	AE_bin104_1	3.45-5.317	7.19	17.3	0.018	Drate_14_all	2.9	14.AllDR1.1	1	20.815	IA_bin3_10	19.78-20.815	5.45	15	-0.034
		14.AllDR6.1	6	34.355	AE_bin257_13	34.27-34.355	5.15	12	0.016			14.AllDR5.1	5	8.274	IA_bin130_22_503_2	8.183-8.274	6.03	16.8	0.031
												14.AllDR6.1	6	19.168	IA_bin324_6	19.095-19.168	5	13.6	-0.031
Drate_17_1	2.6	17.1DR1.1	1	1.911	AE_bin65_2	1.22-4.911	3.58	9.0	0.013										
		17.2DR2.1	2	24.381	AE_bin25_3	24.381-26.318	3.7	9.4	0.014										
Drate_17_2	2.7	17.3DR3.1	3	14.276	AE_bin105_7	8.884-18.276	2.95	6.1	0.017										
		17.2DR4.1*	4	25.997	AE_bin222_330_313_1	25.861-25.997	4.42	9.3	0.02										
		17.2DR6.1*	6	39.593	AE_bin283_1	38.360-39.593	8.67	19.5	0.032										
Drate_17_all	2.6	17.AllDR3.1*	3	24.48	AE_bin108_1	23.745-25.48	3.53	7.4	0.013	Drate_17_all	2.8	17.AllDR1.1	1	21.144	IA_bin4_2	20.815-21.144	2.86	12.4	-0.037
		17.AllDR4.1	4	25.573	AE_bin221_1	25.451-25.573	2.84	5.9	0.011										
		17.AllDR6.1*	6	35.963	AE_bin251_3	35.755-35.963	8.83	20.2	0.022										
Drate_20_1	2.6	20.1DR1.1	1	1.22	AE_bin66_3	0-1.220	4.76	11	0.015										
		20.1DR3.1	3	3.45	AE_bin103_2	2.914-3.45	2.85	6.4	0.012										
		20.1DR6.1*	6	40.185	AE_bin282_3	39.593-42.185	4.56	10.5	0.016										
Drate_20_2	2.5	20.2DR2.1	2	36.599	AE_bin63_260_253_1	36.409-36.599	4.23	7.2	0.03										
		20.2DR4.1*	4	27.573	AE_bin221_1	27.451-27.573	12.88	25	0.042										
		20.2DR6.1*	6	33.384	AE_bin263_50_86_1	33.233-33.384	7.11	12.6	0.031										
Drate_20_all	2.6	20.AllDR2.1	2	36.599	AE_bin63_260_253_1	36.409-36.599	3.66	7.2	0.018	Drate_20_all	2.7	20.AllDR1.1	1	20.814	IA_bin3_10	19.78-20.815	3.63	10.3	-0.036
		20.AllDR4.1*	4	27.573	AE_bin221_1	25.451-25.573	7.28	15.1	0.02			20.AllDR5.1	5	3.063	IA_bin118_4	1.138-4.063	5.05	14.8	0.038
		20.AllDR6.1	6	33.035	AE_bin263_50_86_2	32.851-33.035	6.93	14.2	0.02			20.AllDR6.1	6	25.144	IA_bin370_3	25.015-25.144	4.37	12.6	-0.039

HghtFI _14	2.6	14HTF L1.1*	1	10.534	AE_bin88_525_ 28_2	10.283- 10.534	7.85	11.9	2.03	Height _14	2.9	14HG T2.1	2	31.589	IA_bin177_5	31.055- 31.589	5.26	37.3	-41.16
		14HTF L2.1*	2	17.788	AE_bin28_3	17.473- 17.788	5.87	8.6	2.067										
		14HTF L3.1	3	77.374	AE_bin167_4	77.136- 77.374	6.77	10.1	-1.858										
		14HTF L4.1*	4	27.839	AE_bin206_4	27.494- 27.839	6.82	10.1	1.914										
		14HTF L6.1*	6	32.806	AE_bin229_236 _46_42_97_221	32.730- 32.806	3.54	5.0	1.373										
HghtFI _17	2.5	17HTF L1.1*	1	11.03	AE_bin81_41_2 8_2	10.965-11.03	5.3	5.7	1.3	Height _17	2.8	17HG T2.1	2	47.967	IA_bin238_4	47.499- 47.967	5.54	12.5	-30.12
												17HG T5.1*	5	7.492	IA_bin128_1	7.153-7.492	9.82	25	-47.1
		17HTF L3.1	3	23.745	AE_bin107_2	20.69-23.745	4.98	5.8	-1.3										
		17HTF L4.1*	4	27.839	AE_bin206_4	27.494- 27.839	24.09	37.7	3.359										
		17HTF L6.1*	6	32.544	AE_b259_236_ 46_42_97_222	32.462- 32.544	6.29	7.5	1.549										
HghtFI _20	2.6	20HTF L3.1*	3	66.144	AE_bin149_6	65.848- 66.144	4.81	6.7	-1.392	Height _20	2.8	20HG T2.1*	2	47.344	IA_bin224_62 _288_352_22	47.291- 47.344	5.27	15.6	-36.5
		20HTF L4.1*	4	27.839	AE_bin206_4	27.494- 27.839	26.89	52.8	3.94			20HG T5.1*	5	5.207	138	5.085-5.207	3.68	10.5	-34.7

*QTL identified in at least two replicates with locations within 5 cM and within 5 cM of the QTL detected using the trait average.

Table S9. Summary of QTL for traits unique to the *P. integrifolia* × *P. axillaris* (IA) population.

Trait	LOD threshold	QTL	Chr.	Position (cM)	Nearest Marker	Support interval (1 LOD)	LOD	Explained variance (%)	Additive effects
LNMS_14	2.8	*14LNMS5.1**	5	10.168	IA_bin149_217	10.133-10.168	4.46	24.5	2.35
Branch_14	2.8	*14BR1.1**	1	20.815	IA_bin3_10	19.78-20.815	10.48	38	-2.6
HMS_14	2.8	*14HMS2.1*	2	38.639	IA_bin190_1	37.26-38.639	5.46	19.7	-31.81
		14HMS5.1	5	7.755	IA_bin126_11_526_2	7.492-7.755	3.66	12.4	-24.2
HSB_14	2.8	*14HSB5.1*	5	5.894	IA_bin123_2	5.521-5.894	6.26	32.6	-36
FlBudPS_14	2.6	*14FBP6.1**	6	7.06	IA_bin309_5	15.655	7.06	23.6	18.77
		*14FBP6.2**	6	16.035	IA_bin311_5	16.035	9.12	32	-21.97
FlBud_14	2.8	*14FB1.1*	1	34.171	IA_bin100_5	33.44-34.171	4.45	13.6	-31.61
		14FB2.1	2	29	IA_bin166_3	28.743-29	8.08	27	39.78
		14FB6.1	6	9.202	IA_bin289_1	9.054-9.202	3.88	11.7	-27.52
FlBranchNum_14	2.8	*14FBN1.1**	1	21.144	IA_bin4_2	20.815-21.144	10.99	39.4	-2.63
LNMS_17	2.8	*17LNMS5.1*	5	11.262	IA_bin142_1	11.04-11.262	3.14	31.6	2.47
Branch_17	2.7	*17BR1.1**	1	21.799	IA_bin5_8	21.144-21.799	5.12	21.2	-1.89
HSB_17	2.8	*17HSB5.1*	5	4.348	IA_bin119_24	3.063-4.348	3.66	37.4	-29.1
FlBudPS_17	2.8	*17FBP3_1.1*	3_1	18.276	IA_bin389_21_36_2	17.115-18.276	3.84	12.5	3.71
		17FBP6.1	6	10.176	269/270	10.176	5.78	19.7	-3.71
FlBranchNum_17	2.7	*17FBN1.1**	1	21.799	IA_bin5_8	21.144-21.799	4.69	19.6	-1.84
Branch_20	2.7	*20BR1.1*	1	19.283	11489	18.27-19.283	7.07	24.9	-1.49
HMS_20	2.9	*20HMS2.1*	2	33.959	IA_bin179_2	33.796-33.959	3.12	23.8	-45.51
		20HMS4.1	4	6.146	IA_bin276_1	5.875-6.146	4.13	34.1	-47.1
HSB_20	3	*20HSB2.1*	2	30.487	IA_bin172_1	30.13-30.487	3.16	36.6	-52.8
FlBudPS_20	2.7	*20FBP2.1*	2	16.411	IA_bin394_2	15.653-16.411	3.09	11.4	2.09
		20FBP6.1	6	15.073	IA_bin307_5	14.804-15.073	3.6	13.5	-2.4
FlBud_20	2.7	*20FB5.1*	5	8.274	IA_bin130_22_503_2	8.183-8.274	4.25	15.8	16.53
FlBranchNum_20	2.6	*20FBN1.1*	1	19.283	11489	18.27-19.283	8.29	26.5	-1.51
		20FBN3_2.1	3_2	6.376	IA_bin433_1	5.841-6.376	3.01	8.6	0.74

*QTL identified in at least two replicates with locations within 5 cM and within 5 cM of the QTL detected using the trait average.

Supplementary Table S10. Summary table of QTL rich regions (≥ 5 QTL/cM) in both populations

Population	Chr Number	Region (cM)	SNP number*	Percentage of total SNPs on the same chromosome
AE	2	22.113-22.995	557	45.0%
	3	3.45-4.317	3	0.32%
	4	25.022-25.997<br>27.179-27.839	296.25	34.5%
	6	32.195-33.948	602	74.7%
IA	1	18.3-21.8	40	5.3%
	2	28.7-34.0	54	9.5%
	5	4.1-6.7	46	8.7%

*The number of SNPs per sub-bin was calculated as the number of total SNPs in the bin divided by the number of sub-bins
